# The alpha angle as a predictor of contralateral slipped capital femoral epiphysis

**DOI:** 10.1007/s11832-016-0732-x

**Published:** 2016-04-06

**Authors:** Matthew J. Boyle, Jose F. Lirola, Grant D. Hogue, Yi-Meng Yen, Michael B. Millis, Young-Jo Kim

**Affiliations:** Department of Orthopaedic Surgery, Harvard Medical School, Children’s Hospital Boston, Boston, USA; Department of Pediatric Orthopaedics, Starship Children’s Hospital, Auckland Central, Private Bag 92 024, Auckland, 1142 New Zealand; Department of Pediatric Orthopaedics, Hospital Universitario Virgen del Rocío, Seville, Spain; Department of Pediatric Orthopaedics, University of Texas Health Science Center at San Antonio, San Antonio, TX USA

**Keywords:** Slipped capital femoral epiphysis, Alpha angle, Hip, Fixation

## Abstract

**Purpose:**

Contralateral hip involvement in slipped capital femoral epiphysis (SCFE) is common. Femoral head−neck asphericity, as measured by an elevated alpha angle, has not previously been assessed with respect to SCFE risk. Our aim was to assess the utility of the alpha angle in predicting contralateral SCFE.

**Methods:**

We retrospectively reviewed 168 patients (94 males) managed surgically for unilateral SCFE between 2001 and 2013 who had a minimum of 18 months follow-up. The alpha angle, the posterior sloping angle (PSA), and the modified Oxford score were recorded for every patient at the time of initial SCFE presentation. Follow-up clinical records and radiographs were assessed to determine the presence of absence of contralateral SCFE.

**Results:**

Forty-five patients (27 %) developed a contralateral SCFE. Patients who developed a contralateral SCFE had a significantly higher alpha angle (51° vs 45°, *p* < 0.001) than patients who did not develop a contralateral SCFE. There was no significant difference in PSA or modified Oxford score (both *p* > 0.10) between patients who developed a contralateral SCFE and those who did not. Using a proposed alpha angle of 50.5° as a threshold for prophylactic fixation, 26 (58 %) of the 45 cases of contralateral SCFE in our study would have been prevented and 18 (15 %) of 123 patients would have undergone fixation unnecessarily.

**Conclusions:**

We found the alpha angle to positively correlate with contralateral SCFE risk. Patients with significantly elevated alpha angles may be at greater risk of contralateral SCFE and benefit from further investigation or prophylactic hip fixation.

## Introduction

Slipped capital femoral epiphysis (SCFE) is a common adolescent hip disorder with a varied international incidence [1, 2]. The reported frequency of bilateral slip varies markedly from approximately 16–60 % [3, 4], with the highest incidence of 80 % reported by Billing and Severin [5] using an advanced radiographic technique. In >88 % of cases, the contralateral slip occurs within 18 months after the initial SCFE [6, 7]. Prophylactic fixation may prevent deformity and future secondary degeneration in certain patients; however, surgery on a hip that may never develop pathology is controversial [8].

A number of SCFE risk factors have been proposed, including younger age [9, 10], obesity [11], renal insufficiency [12], endocrine abnormalities such as hypothyroidism and growth hormone deficiency [13], and ethnicity [10]. Abnormal mechanical forces acting across the capital femoral physis are also likely to play an important role [14]. Relative or actual femoral neck retroversion [15], capital femoral physeal orientation [16, 17], and changes in physeal or periphyseal strength [18] have all been implicated as potential mechanical causes of SCFE.

Femoral head–neck asphericity is another mechanical phenomenon that may contribute toward capital physeal instability in SCFE patients. Patients with femoral head–neck asphericity, as measured by an elevated alpha angle of Notzli et al. [19], are predisposed toward cam-type femoroacetabular impingement [20]. Theoretically, the repetitive femoroacetabular contact that occurs in these patients may result in physeal instability; however, the alpha angle has not previously been assessed with respect to SCFE risk. The purpose of this study was to assess the utility of the alpha angle in predicting contralateral SCFE.

## Materials and methods

This was a retrospective cohort study investigating the relationship between the alpha angle and rate of contralateral SCFE in patients managed surgically for unilateral SCFE.

Following institutional review board approval, we identified 420 patients treated surgically for unilateral SCFE at our institution between June 2001 and September 2013 through a review of hospital records. We excluded patients with <18 months follow-up (*n* = 127), patients with incomplete initial plain radiography (*n* = 51), patients who underwent prophylactic fixation of the contralateral hip (*n* = 38), and patients who had contralateral hip pain on presentation (*n* = 36). Although none of the excluded patients with contralateral hip pain were subsequently diagnosed with SCFE, these patients were excluded from the analysis due to the possibility of missed or undiagnosed SCFE and in order to focus on the risk of subsequent SCFE after unilateral slip. These exclusions left 168 patients (94 male, 74 female), with a mean age at initial presentation of 12.2 years (range 8.6–16.8 years), a mean body mass index (BMI) at initial presentation of 26.9 kg/m^2^ (range 15.2–47.5 kg/m^2^), and a mean follow-up of 44 months (range 18–142 months) to be included in the analysis (Table 1). Patients described their ethnicity as Caucasian (*n* = 99; 59 %), African American (*n* = 31; 19 %), Hispanic (*n* = 12; 7 %), or other (*n* = 24; 14 %). Five patients (3 %) had a documented endocrine abnormality. The majority of patients presented with a stable SCFE as defined by Loder et al. (stable *n* = 131; 78 %/unstable *n* = 37; 22 %). Patients were treated with in situ pinning (*n* = 146; 87 %), open reduction (*n* = 13; 8 %), or osteotomy (*n* = 9; 5 %).

**Table 1:** Patient information at the time of presentation with initial SCFE

	All patients	Contralateral SCFE	No contralateral SCFE	Odds ratio^a^ (95 % CI)	*p* value^a^
No. of patients	168	45	123		
Patient age (years)^b^	12.2 ± 1.52	12.2 ± 1.50	12.2 ± 1.53	0.90 (0.61–1.34)	0.84
Gender male^c^	94 (0.56)	26 (0.58)	68 (0.55)	1.30 (0.41–4.17)	0.78
BMI (kg/m^2^)	26.9 ± 5.95	26.9 ± 5.53	26.9 ± 6.12	0.96 (0.88–1.05)	0.98
Endocrine abnormality^c^	5 (0.03)	3 (0.07)	2 (0.02)	1.99 (0.19–20.93)	0.12
SCFE unstable^c^	37 (0.22)	8 (0.18)	30 (0.24)	0.49 (0.15–1.60)	0.43

*CI* confidence interval, *BMI* body mass index

^a^Comparison of mean or proportion between patients who did and did not develop contralateral SCFE

^b^Mean ± standard deviation

^c^No. of patients (proportion of group)

**Fig. 1:**
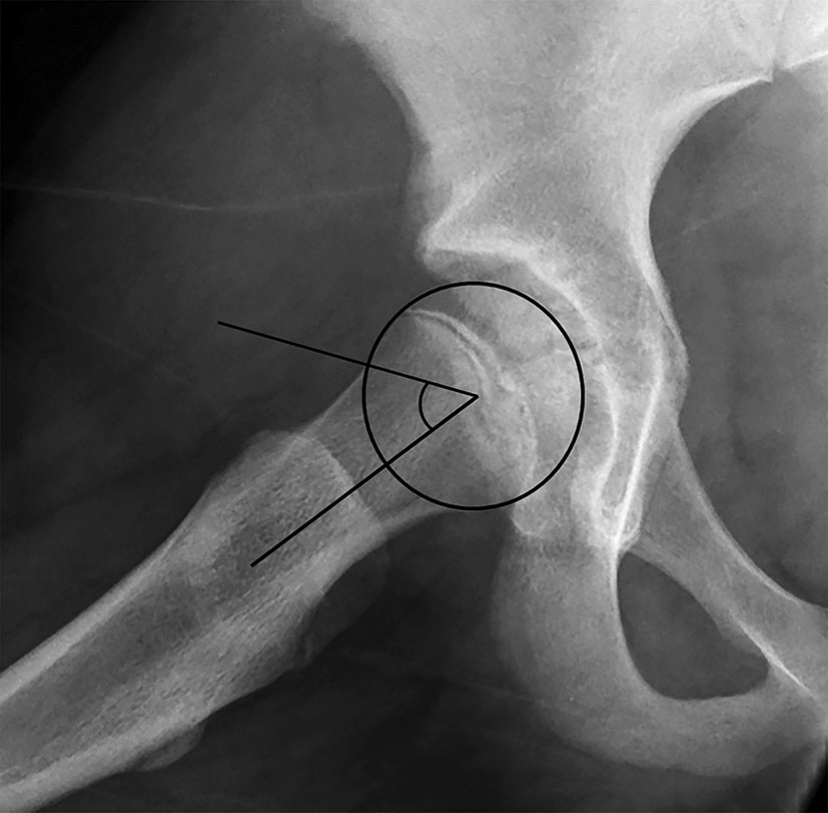
Frog-leg lateral radiograph of the asymptomatic right hip of an 11-year-old female presenting with left SCFE, demonstrating an alpha angle of 54°. The alpha angle is measured by placing a perfect *circle* over the femoral head and measuring the angle formed between a *line* from the center of the femoral head to the center of the femoral neck and a *second line* from the center of the femoral head to the point at which the anterior femoral neck leaves the perfect circle

All radiographs were reviewed by the primary author, a pediatric orthopedic surgery fellow, who was blinded to the presence or absence of contralateral SCFE. The alpha angle, the posterior sloping angle (PSA), and the modified Oxford score were recorded for each patient at the time of initial SCFE presentation. The alpha angle and PSA were calculated from the asymptomatic contralateral hip according to the methods described by Notzli et al. [19] and Barrios et al. [21], respectively, using frog-leg lateral radiographs (Fig. 1). Frog-leg lateral radiographs are performed routinely at our institution with the patient supine, with feet together and hips abducted as widely as tolerated. The alpha angle was measured by first placing a best fit circle over the femoral head. One arm of the alpha angle was then placed, which was drawn extending along the long axis of the femoral neck from the center of the femoral neck at its narrowest point to the center of the best fit circle. The second arm of the alpha angle was then placed, which was drawn from the center of the best fit circle to the point anteriorly where the femoral head or neck extends beyond the margin of the circle. The alpha angle thus formed provides a quantitative measurement of the degree of femoral head asphericity, or lack of femoral head−neck junction concavity (Fig. 1). The PSA was measured by drawing three lines. The first line is drawn from the center of the proximal femoral shaft through the center of the proximal femoral metaphysis, representing the longitudinal axis of the femur. The second line is drawn from one edge of the proximal femoral physis to the other, representing the physeal axis. Where these two lines intersect, a third line is drawn perpendicular to the first line. The PSA is the angle formed by the second and third lines, with an increasing angle correlating with more posterior orientation of the proximal physis. The modified Oxford score [22] is a measure of bone age that correlates with physiological maturity, that can be obtained from routine plain radiographs of the hips and pelvis. The modified Oxford score was calculated from the asymptomatic contralateral hip according to the methods described by Stasikelis et al. [23] using anteroposterior radiographs to evaluate the skeletal maturity of the head of the femur, the greater trochanter, and the ilium, and frog-leg lateral radiographs to evaluate the skeletal maturity of the lesser trochanter and the triradiate cartilage. As described by Stasikelis et al. [23], each area was scored within a range of two or three points according to advancing bony and physeal maturity—5, 6, or 7 points for the head of the femur; 4, 5, or 6 points for the greater trochanter; 3 or 4 points for the ilium; 3, 4, or 5 points for the lesser trochanter; and 1, 2, or 3 points for the triradiate cartilage, for a possible total score of 16–25 with a higher score indicating more advanced skeletal maturity. Half points were not awarded.

The radiographs of a subset of ten patients randomly selected using a random number generator were reviewed by two additional observers (a pediatric orthopedic surgeon and an additional pediatric orthopedic surgery fellow) to permit interobserver reliability calculation, and by the primary author at one month after the initial analysis to permit intraobserver reliability calculation. All observers were blinded to the presence or absence of contralateral SCFE. Interobserver reliability was excellent for the alpha angle (intraclass correlation coefficient [ICC] 0.92; 95 % CI 0.78–0.98), PSA (ICC 0.79; 95 % CI 0.34–0.95), and modified Oxford score (ICC 0.83; 95 % CI 0.46–0.96). Intraobserver reliability was excellent for the alpha angle (ICC 0.92; 95 % CI 0.70–0.98) and modified Oxford score (ICC 0.88; 95 % CI 0.55–0.97), and fair for the PSA (ICC 0.46; 95 % CI 0.32–0.85). ICCs were interpreted according to the criteria of Fleiss [24] and Cicchetti and Sparrow [25] as <0.40 = poor, 0.40–0.59 = fair, 0.60–0.74 = good, >0.74 = excellent.

The clinical records and radiographs of all included patients were assessed to determine the presence or absence of a contralateral SCFE, based on whether in situ screw fixation was performed on the contralateral side.

### Statistical analyses

Continuous characteristics that met the assumptions of normality were summarized by mean and standard deviation and compared across groups using Student’s *t* test. For continuous characteristics that deviated from normality, data were summarized by median and interquartile range (25th–75th percentile) and compared across groups using the Mann–Whitney *U* test. Binary characteristics were summarized by frequency and percent and compared across groups using chi-squared test. Univariate and multivariable binary logistic regression were used to identify potential risk factors of contralateral SCFE in subjects presenting with unilateral SCFE. Factors analyzed included age at presentation, gender, BMI, incidence of endocrine disorder, clinical SCFE stability, alpha angle, PSA, and modified Oxford score. Model fit was assessed using Akaike’s information criteria and the likelihood ratio test. Odds ratios along with 95 % CIs were estimated for significant factors. Based on significant risk factors, receiver operating characteristic (ROC) analysis was implemented to assess the ability of factors to detect contralateral SCFE in patients presenting with unilateral SCFE. The area under the ROC curve was estimated along with a 95 % CI. For continuous risk factors an optimal cut-off value was calculated based on Youden’s index (identifies the point on the ROC curve that simultaneously maximizes sensitivity and specificity). The Pearson product-moment correlation coefficient was employed as a measure of linear correlation between alpha angle and PSA results, with a correlation coefficient from 0−0.25 defined as an absence of correlation, 0.25–0.5 indicating poor correlation, 0.5–0.75 indicating good correlation, and 0.75–1 indicating excellent correlation between variables, as defined by Dawson and Trapp [26]. All tests were two-sided and *p*-values <0.05 were considered significant. Analyses were conducted using SAS version 9.3 (SAS Institute Inc., Cary, NC, USA).

## Results

Forty-five patients (27 %) developed a contralateral SCFE during the study period, at a mean of 10.3 months (range 1.1–46.6 months) after initial SCFE. Patients who developed a contralateral SCFE had a significantly higher alpha angle (51° vs 45°, *p* < 0.001) than patients who did not develop a contralateral SCFE (Table 2). There was no significant difference in age (*p* = 0.84), gender (*p* = 0.78), ethnicity (*p* = 0.86), BMI (*p* = 0.98), incidence of endocrine abnormality (*p* = 0.12), initial SCFE stability (0.43), PSA (*p* = 0.11) or modified Oxford score (*p* = 0.50) between patients who developed a contralateral SCFE and those who did not (Tables 1, 2).

**Table 2:** Radiographic parameters at the time of presentation with initial SCFE

	All patients	Contralateral SCFE	No contralateral SCFE	Odds ratio^a^ (95 % CI)	*p* value^a^
No. of patients	168	45	123		
Alpha angle (°)^b^	46.5 ± 6.89	50.6 ± 8.83	44.9 ± 5.29	1.10 (1.03–1.18)	<0.001
PSA (°)^b^	13.0 ± 7.23	15.0 ± 7.79	12.2 ± 6.9	1.05 (0.99–1.12)	0.11
Modified Oxford score^c^	19 (18–20)	19 (17–20)	19 (18–20)	0.99 (0.71–1.34)	0.50

*CI* confidence interval, *PSA* posterior sloping angle

^a^Comparison of mean or median between patients who did and did not develop contralateral SCFE

^b^Mean ± standard deviation

^c^Median (interquartile range)

Of the patients who did develop a contralateral SCFE, there was no significant correlation between alpha angle and time to contralateral slip (*p* = 0.12) or modified Oxford score and time to contralateral slip (*p* = 0.34). There was a significant negative correlation between PSA and time to contralateral slip (correlation coefficient −0.48 (95 % CI −0.68 to −0.21); *p* = 0.001); for each one degree increase in sloping angle, the time to slip decreased by 5 %.

Multivariable analyses found the alpha angle to be the only independent risk factor for contralateral SCFE (*p* = 0.004) in our study. For each one degree increase in alpha angle, the relative odds of a contralateral SCFE increased by 10 %. Patient age, gender, BMI, incidence of endocrine abnormality, initial SCFE stability, PSA, and modified Oxford score had no independent significant effect (all *p* > 0.10) on rate of contralateral SCFE. There was no significant correlation between the alpha angle and PSA (correlation coefficient 0.22).

**Fig. 2:**
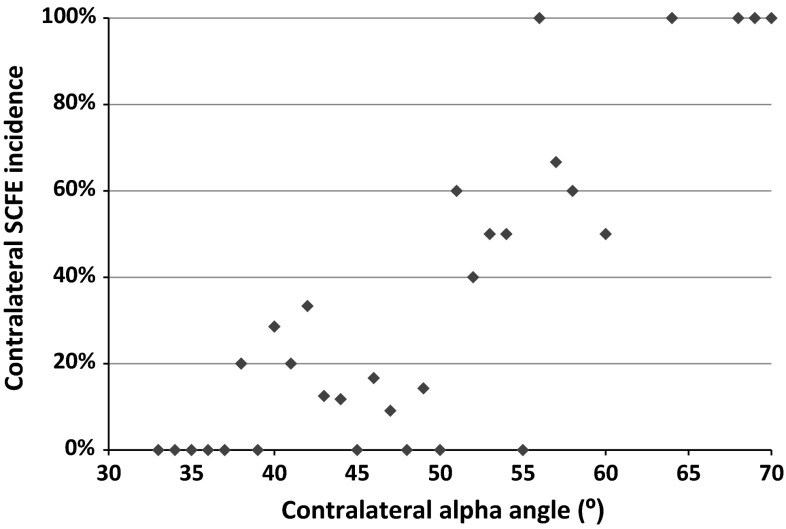
Incidence of contralateral SCFE according to contralateral alpha angle at the time of initial SCFE presentation

The alpha angle had an area under the ROC curve of 0.68 (95 % CI 0.57–0.79), indicating that 68 % of patients who developed a contralateral SCFE had a higher alpha angle than a patient who did not develop a contralateral SCFE. The ROC curve identified an alpha angle of 50.5° as the optimum value to simultaneously optimize sensitivity and specificity; this value had a sensitivity of 58 % and a specificity of 85 % for identifying a patient who would develop a contralateral SCFE. Using an alpha angle of 50.5° as a threshold for prophylactic fixation, 26 (58 %) of the 45 cases of contralateral SCFE in our study would have been prevented and 18 (15 %) of 123 patients would have undergone fixation unnecessarily (Fig. 2).

## Discussion

Orthopedic surgeons must contemplate a multitude of factors when considering prophylactic fixation in SCFE patients. Patient reliability, the intrinsic risks of exposure to an additional surgical procedure with potential complications such as osteonecrosis and chondrolysis, and the risk of contralateral SCFE occurring if prophylactic fixation is not employed with the potential of subsequent associated osteoarthritis, are all important factors.

Our results suggest that patients with femoral head–neck asphericity are at greater risk of developing contralateral SCFE than patients with normal femoral morphology. The precise nature of this relationship is unclear. One possibility is that repetitive subclinical contact between the femoral head−neck junction and acetabulum (femoroacetabular impingement; FAI) occurs in patients with an elevated alpha angle, resulting in increased transphyseal mechanical stresses and leading to eventual symptomatic SCFE. Similar abnormal mechanical contact may also occur in patients with relative or actual femoral neck retroversion, who have previously been identified as being at an increased risk of developing SCFE [27]. On the contrary, a deep acetabulum has not been found to increase contralateral SCFE risk [14], despite acetabular retroversion, coxa profunda, and acetabular protrusio being commonly observed in SCFE patients [14, 28] and known to predispose to FAI [19].

Another possibility is that the contralateral femoral head−neck asphericity seen in our study population represented an asymptomatic mild SCFE in certain patients. While a significant proportion of these patients did later develop a symptomatic SCFE, initial asymptomatic SCFE development is possible. Previous authors have identified relatively high rates of asymptomatic SCFE. In 1996, Jerre et al. found that 42 of 59 patients who developed a contralateral SCFE did so without symptoms [29]. In 2013, Lehmann et al. found that 6.6 % of 2,072 healthy young adults had radiological findings consistent with a prior SCFE [30], suggesting that asymptomatic SCFE may be relatively common. It is important to note, however, that we did not observe a significant correlation between alpha angle and PSA results, suggesting that the elevated alpha angles seen in our series may not be due to incipient slippage of the capital epiphysis. Whether representing a predisposing mechanical environment or a subtle asymptomatic SCFE, our findings suggest that femoral head–neck asphericity correlates with symptomatic contralateral SCFE development and may be useful when considering prophylactic fixation.

An interesting secondary finding was that the average alpha angle of our overall SCFE population was similar to that previously reported in a normal adolescent population [31], which is reassuring with respect to FAI risk. While FAI is relatively common after SCFE due to post-slip femoral deformity [32], it remains unclear whether the contralateral non-slipped hip in SCFE patients is entirely normal. It is important to note that SCFE patients frequently display bilateral acetabular retroversion [8] and increased acetabular depth [14], which may predispose these patients to FAI even in the setting of normal femoral morphology. We feel that it is important to clinically monitor the contralateral hip in SCFE patients, even in the absence of contralateral slip, in order to diagnose FAI early in these susceptible patients. A more accurate measure of the risk of contralateral FAI in unilateral SCFE patients may be the beta angle of Wyss et al. [33]; however, this requires specific hip flexion radiographs [34] or open chamber magnetic resonance imaging [33], which had not been undertaken in our patients.

While the PSA, modified Oxford score, and BMI did not reliably predict contralateral SCFE in our study, we did find a significant correlation between the PSA and time to contralateral SCFE. Previous authors have found these measures to correlate with SCFE incidence. Zenios et al. [35], Park et al. [36], and Phillips et al. [17] identified a PSA of >14.5°, >12.7°, and >14°, respectively, to predict contralateral SCFE. Barbieri et al. [37] and Popejoy et al. [9] found that lower Oxford scores correlated with higher rates of contralateral SCFE. Nasreddine et al. [11] found that obese SCFE patients had a higher risk of bilateral SCFE, and that obese patients who became non-obese postoperatively had a decreased risk of contralateral SCFE; however, they also found that patient age and slip angle were not associated with bilateral SCFE. This discrepancy between SCFE study findings highlights that risk factors should not be considered absolute or used in isolation. We feel that it is important for orthopedic surgeons to consider all available information, including radiographic measurements such as the alpha angle, and undertake shared decision-making when considering prophylactic fixation, considering both outcome probabilities and patient preferences.

Our study has a number of limitations. First, the retrospective nature of the study carries the risk of selection bias; patients included in our study may not be reflective of the general population of SCFE patients. Second, follow-up was relatively short which may have falsely decreased our rate of contralateral SCFE. Previous authors have found that at least 88 % of cases of contralateral SCFE occur within 18 months of the initial SCFE [6, 7]; by utilizing a minimum follow-up of 18 months, we hoped to capture the majority of contralateral SCFE cases. Third, skeletal maturity was determined using the modified Oxford score; however, the use of the Greulich and Pyle radiographic atlas method [38] may have provided a more accurate assessment of skeletal maturity. Fourth, the alpha angle was measured from frog-leg lateral radiographs only. Including measurements taken from additional radiographic views may have strengthened our conclusions; however, the frog-leg lateral view has previously been shown to demonstrate the greatest difference in alpha angle between hips with impingement and control hips [39], and is widely used in the radiographic analysis of SCFE patients [35]. Fifth, the alpha angle may be prone to significant measurement variation. Although our interobserver and intraobserver reliabilities were acceptable, all measurements were performed by fellowship-trained pediatric orthopedic surgeons who were experienced in measuring the alpha angle; however, this reliability may not be applicable to all orthopedic surgeons. Sixth, due to the retrospective nature of the study, contralateral screw fixation was used as a surrogate for symptomatic contralateral SCFE, which does confer the potential of diagnostic error. Finally, we had a relatively high rate of lost to follow-up, illustrating the intrinsic difficulties of achieving close follow-up of SCFE patients [17]. It is important to note that with lost to follow-up and patient exclusions, only 168 patients of 420 initially assessed were included in the study, which may significantly influence the alpha angle threshold and study conclusions.

To our knowledge, we have undertaken the first analysis of the alpha angle with respect to SCFE risk. We found the contralateral alpha angle to positively correlate with contralateral SCFE incidence. Patients with an alpha angle ≥50.5° may be at greater risk of contralateral SCFE and benefit from further investigation or prophylactic hip fixation.
